# Apologies

**Published:** 1854-11

**Authors:** 


*Apologies.*—The large amount of original matter for this month, excludes our eclectic department, and encroaches materially upon the editorial space. The character of the original matter is such, however, as to render our apology scarcely necessary. Dr. Newman's paper on Congestion of the Brain in Cholera, possesses great interest and originality. We hope that those of our readers who are in the habit of passing by, unread, all articles on cholera, will suspend the rules for this occasion, and give their careful attention to this memoir. Dr. N's opportunities for observing cholera have been, (owing to his official position,) much greater than those of any other of our physicians. He was fortunate, also, in being able to control the treatment, his patients being in the hospital and free from interference of officious friends.

The paper on Erysipelas describes very faithfully the same malignant type of disease which occurred in 1844 and '5. If it follows the same course it then pursued, we shall have it further west during the coming winter. H.

